# Exploring the Molecular Crosstalk between Pancreatic Bud and Mesenchyme in Embryogenesis: Novel Signals Involved

**DOI:** 10.3390/ijms20194900

**Published:** 2019-10-03

**Authors:** Ilaria Guerriero, Maria Teresa De Angelis, Fulvio D’Angelo, Rita Leveque, Eleonora Savignano, Luca Roberto, Valeria Lucci, Pellegrino Mazzone, Simona Laurino, Giovanni Storto, Anna Nardelli, Alessandro Sgambato, Michele Ceccarelli, Mario De Felice, Elena Amendola, Geppino Falco

**Affiliations:** 1 Istituto di RicercheGenetiche G. Salvatore, Biogems.c.ar.l, ArianoIrpino, 83031 Avellino, Italy; ilaria.guerriero.ig@gmail.com (I.G.); mariateresadeangelis211285@gmail.com (M.T.D.A.); fulvio.dan13@gmail.com (F.D.); savignanoeleonora@gmail.com (E.S.); luca.roberto@biogem.it (L.R.); 2 Dipartimento di Biologia, Universita’ degliStudi di Napoli, Federico II, 80126 Napoli, Italy; levequerita@gmail.com (R.L.); valeria.lucci@unina.it (V.L.); pellegrinomazzone@gmail.com (P.M.); 3 IRCCS—Referral Cancer Center of Basilicata (CROB), 85028 Rionero in Vulture (PZ), Italy; simona.laurino@crob.it (S.L.); giosto24@hotmail.com (G.S.); alessandro.sgambato@unicatt.it (A.S.); 4 Istituto di Biostrutture e Bioimmagini-CNR, Via De Amicis No. 95, 80145 Napoli, Italy; anna.nardelli@ibb.cnr.it; 5 Department of Science and Technology, University of Sannio, 82100 Benevento, Italy; m.ceccarelli@gmail.com; 6 Istituto per l’Endocrinologia e l’OncologiaSperimentale “G. Salvatore”, CNR, 80131 Napoli, Italy; mario.defelice@unina.it

**Keywords:** pancreatic stem cells, pancreatic disorders, embryonic stem cells, bud, progenitor cells, laser microdissection, mesenchymal stem cell

## Abstract

Pancreatic organogenesis is a multistep process that requires the cooperation of several signaling pathways. In this context, the role of pancreatic mesenchyme is important to define the epithelium development; nevertheless, the precise space–temporal signaling activation still needs to be clarified. This study reports a dissection of the pancreatic embryogenesis, highlighting the molecular network surrounding the epithelium–mesenchyme interaction. To investigate this crosstalk, pancreatic epithelium and surrounding mesenchyme, at embryonic day 10.5, were collected through laser capture microdissection (LCM) and characterized based on their global gene expression. We performed a bioinformatic analysis to hypothesize crosstalk interactions, validating the most promising genes and verifying the precise localization of their expression in the compartments, by RNA in situ hybridization (ISH). Our analyses pointed out also the c-Met gene, a very well-known factor involved in stimulating motility, morphogenesis, and organ regeneration. We also highlighted the potential crosstalk between Versican (Vcan) and Syndecan4 (Sdc4) since these genes are involved in pancreatic tissue repair, strengthening the concept that the same signaling pathways required during pancreatic embryogenesis are also involved in tissue repair. This finding leads to novel strategies for obtaining functional pancreatic stem cells for cell replacement therapies.

## 1. Introduction

Cell replacement therapies in treatments for pancreatic injury, such as diabetes and pancreatitis, have the critical point of the limited availability of pancreatic progenitor cells (PPCs). Functional pancreatic cells can be generated through different procedures: from embryonic stem cells (ESCs) or induced pluripotent stem cells (iPSCs) differentiation, from adult stem cells of pancreatic ducts and islets, by transdifferentiation of other pancreatic cell types [1,2]. Although new knowledge about signals essential for pancreatic organogenesis has been discovered, the crucial limit is still the lack of information about the regulation of the key molecular pathways of pancreatic morphogenesis, in order to improve efficiency and quality of PPCs derived by differentiation protocols [3]. The final goal is to mimic the in vivo pancreatic ontogenesis enhancing the in vitro signals required for PPC specification [4].

In mice, pancreatic organogenesis is a multistep progression made up of three stages: primary regulatory transition, from embryonic day 8.5 to embryonic day 10.5 (E8.5–E10.5), secondary regulatory transition (E13.5–E16.5), and third regulatory transition (after birth) [5]. The primary regulatory transition is the key time point for pancreas development: at E10.5 the organ determination is decided, defined as the conversion of predifferentiated cells to a proto-differentiated state [6]. Molecular mechanisms that regulate the primary transition have been mainly focused on bud intrinsic signals [3], although also mesenchymal extrinsic signals play an important role: the interaction between pancreatic bud intrinsic signals and mesenchyme extrinsic signals is defined as crosstalk. Golosow and Grobstein revealed, for the first time, the concept of crosstalk during morphogenesis, showing that mesenchyme-free pancreatic epithelium failed to grow and differentiate [7]. Recently, the discovery that endothelial factors coming from the aorta initiate pancreatic epithelium formation can explain that, until E9.5, vasculogenesis is the leading biological process that inducts pancreas development; at E10.5, the dorsal pancreatic epithelium forms a proper bud and after the separation of the aorta from the mesenchyme layer, mesenchymal signals regulate pancreatic organogenesis through the successive embryonic stages [8].

The signals that govern pancreatic epithelium–mesenchyme crosstalk are retinoic acid (RA), Wnt, Hedgehog, FGF, BMP, TGF-β, and EGF [9]. Fgf10, a ligand for FGFR2b detected in the epithelium, is expressed by pancreatic mesenchyme and it is required between E9.5 and E11.5 for proliferation of pancreatic epithelial progenitor cells [10,11]. At the same time, Wnt signaling must be inhibited in the early endoderm for pancreas specification; in fact, constitutive activation of the Wnt pathway causes a loss of Fgf10 expression in the mesenchyme [12]. In addition, the Wnt signaling activation is strictly required for pancreas specification, since it has been demonstrated that the ectopic activation of this pathway leads to the gastric and intestinal aberrant induction in the pancreatic epithelium and mesenchyme [13]. Furthermore, Raldh2, an enzyme of retinoic acid biosynthesis, is expressed in the splanchnic mesoderm that surrounds the early budding pancreatic epithelium, thus RA can promote the outgrowth of the dorsal pancreatic bud and the formation of the early endocrine cells [14,15]. Similarly, BMP and Activin from the splanchnic mesoderm are required for proper specification of the early ventral pancreas [16]. Still, TGF-β and Notch signaling may act in parallel pathways to control pancreatic endocrine cell progenitor expansion, although Notch controls cell fate on reciprocal signaling between adjacent cells in the epithelium and is active in the early pancreatic epithelium [17]. Altogether, this evidence shows that a fine cooperation among these signaling pathways is needed for pancreatic organogenesis [18].

The lack of knowledge about how signaling pathways that determine pancreatic epithelium formation work individually and in combination prompted us to characterize the molecular network that contributes to the pancreatic crosstalk. Since E10.5 is the fundamental time point at which cells proliferate without either differentiating or initiating epithelial-to-mesenchymal transition, we have exploited laser capture microdissection (LCM) technologies to collect the pancreatic bud and surrounding mesenchyme and characterized their global gene expression profile to discover new molecules involved in pancreatic crosstalk.

## 2. Results

### 2.1. Global Gene Expression Profiling between Dorsal Pancreatic Bud and Surrounding DPB Mesenchyme

**Figure 1:**
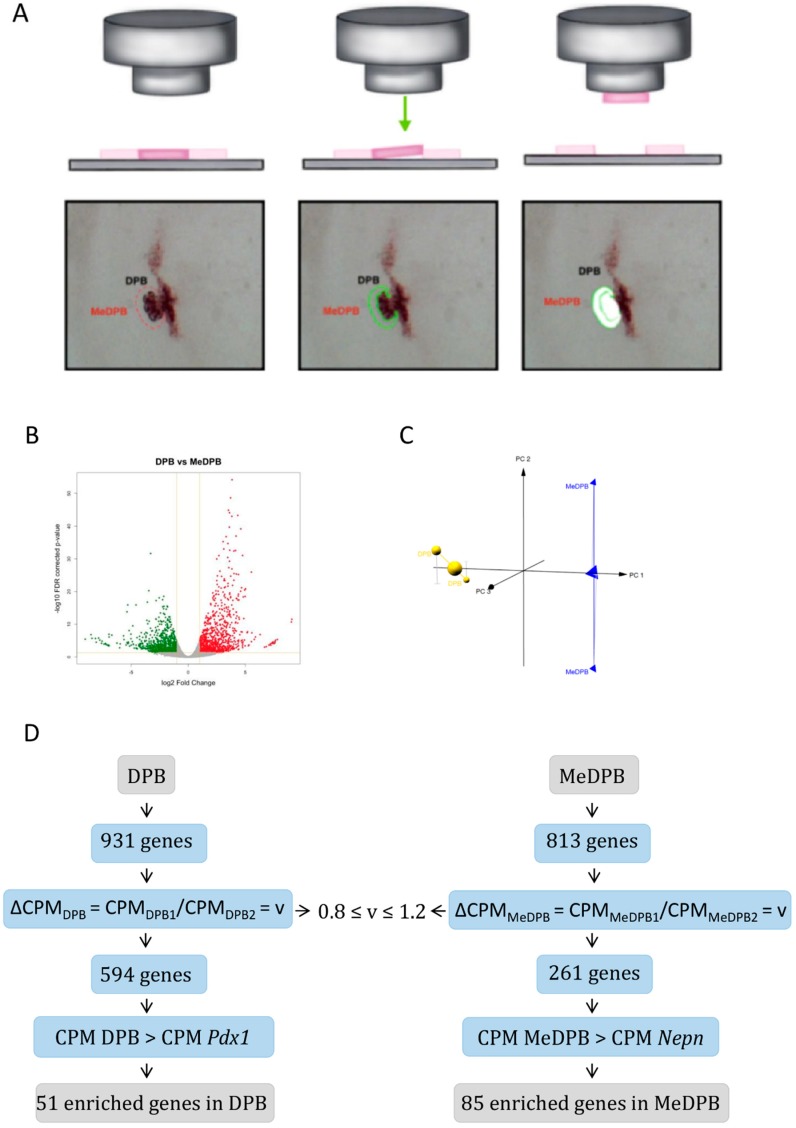
Capture of dorsal pancreatic bud and dorsal pancreatic mesenchyme by Laser Capture Microdissection. (**A**) Schematic representation of DPB and MeDPB dissection. Samples were collected by using the LCM technology at the embryonic stage E10.5. To reduce the biological variability, DPB and MeDPB from three embryos were collected and pooled. (**B**) Volcano plot of differentially expressed genes. Each dot on the plot is a single gene feature. Horizontal axis: fold change (in log2 scale); vertical axis: FDR-corrected p-value (in log10 scale). Color coding is based on the fold change. Thick vertical lines highlight fold changes of −2 and +2, while a thick horizontal line represents a *p*-value of 0.05. (**C**) PCA plot. Samples were represented in three-dimensional PCA plot. (**D**) Flowchart adopted to identify enriched genes in DPB (left panel) and MeDPB (right panel). According to RNA-seq analysis among 1744 genes significantly differentially expressed (absolute log2 Fold Change ≥ 1 and FDR corrected *p*-value ≤ 0.05), 931 and 813 genes were specifically highly expressed in DPB and in MeDPB, respectively. We consider genes based on their low variability (v) between replicates (0.8 ≤ v ≤ 1.2 about 20% in the two conditions) and a cutoff of CPM value of Pdx1 gene as a positive expressed control for DPB genes and a CPM value of Nepn gene as a negative control for the MeDPB genes.

In order to characterize the molecular expression profiles, we dissected mouse dorsal pancreatic bud (DPB) and DPB mesenchyme (MeDBP) by LCM at E10.5 (Figure 1A) and analyzed the gene expression profile by RNA high-throughput sequencing (RNAseq). Considering a cutoff of Log_2_ Fold Change ≥ 1 and FDR-corrected *p*-value ≤ 0.05, a total of 1744 significantly differentially expressed genes were identified, as reported in a volcano plot (Figure 1B). Global gene expression was explored, reducing the high dimensionality of the data (thousands of variables) in few components by principal component analysis PCA [19]. Samples were represented in a three-dimensional PCA plot (Figure 1C) to assess their variability. The strong separation of DPB from MeDPB replicates in the PCA plot confirmed the specific gene expression profiles that molecularly characterized the two biological districts. Among these 1744 differentially expressed genes, 931 were specifically enriched in DPB and 813 genes were specifically enriched in MeDPB (Table S1).

To identify specific markers of pancreatic bud and mesenchyme, the gene expression profiles of murine DPB and MeDPB at E10.5 were analyzed. To quantify transcripts, our analysis was primarily based on the number of reads that mapped to each transcript sequence. To reduce the variability derived from sequencing performance, read counts were normalized for library size (millions of reads) resulting in counts per millions (CPM). We focused our attention on CPM values useful to measure the expression level of a gene. Among the 1744 differentially expressed genes, we considered the top 50 genes based on replicates variability, with at most 20% in the two conditions, and cutoff of CPM value of the Pdx1 gene as a positive expressed control for DPB genes and a CPM value of the Nepn gene as a negative control for the MeDPB genes (Figure 1D).

In Table 1 and Table 2 are reported the top 50 genes enriched in DPB and MeDPB, respectively, ordered by CPM value.

The expression of genes known in literature as epithelial pancreatic markers, such as Nepn, Sox9, Notch1, Gcg, and Pdx1 [3,20,21,22] in the DPB list demonstrated RNA-seq analysis quality.

**Figure 2:**
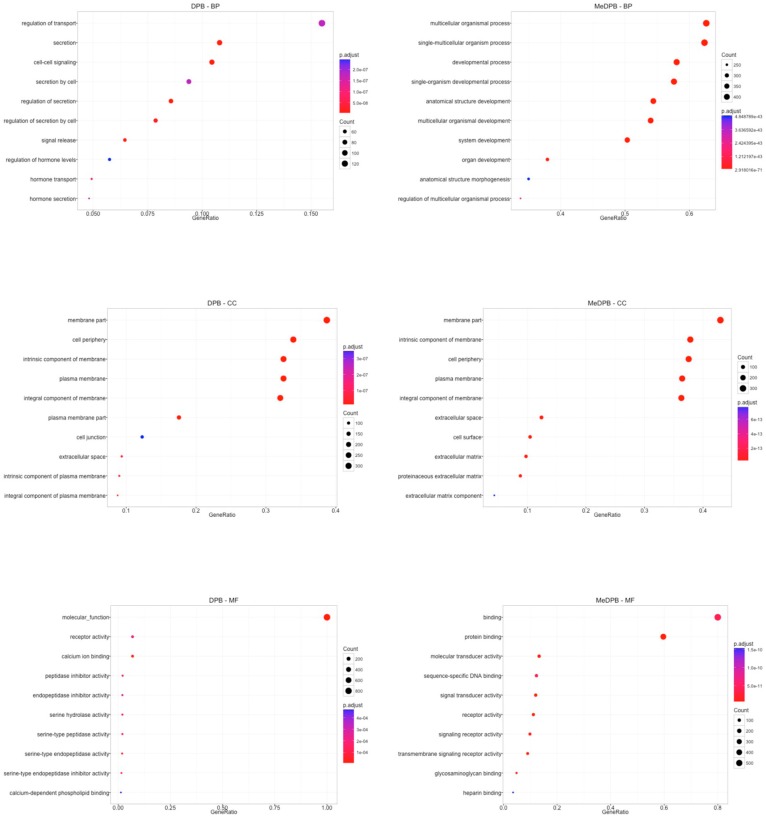
Gene Ontology analysis. The functional characterization of the specific gene expression profiles identified in the two compartments, DPB and MeDPB, was achieved by the annotation of differentially expressed genes with Gene Ontology (GO) terms including Biological Processes (BP), Cellular Components (CC), and Molecular Functions (MF). Over-representation test and False Discovery Rate (FDR) correction were performed to identify the most significantly enriched GO terms in DBP and MeDPB. In the dot plots, the top significant terms (defined as the ten with the lowest FDR-adjusted *p*-value) were reported on the y-axes, whereas gene ratio (defined as the fraction of overlapping genes) were indicated on the x-axes. Gene count (defined as the number of overlapping genes) and FDR-adjusted *p*-value were also represented in the plots by dot size and color, respectively.

To understand the biological meaning of observed gene expression differences, we performed a functional enrichment analysis to identify significantly represented Gene Ontology terms (GO). In particular, DPB and MeDPB were characterized by the expression of specific genes included in Biological Process terms that discriminate the two compartments (Figure 2 and Tables S2 and S3).

### 2.2. In Vitro and In Vivo Characterization of Enriched Intrinsic Factors

To identify DPB- and MeDPB-specific markers, we further analyzed the differentially expressed genes based on their Expressed Sequence Tag (EST) profile of these candidates [23]. We chose genes that showed an annotated expression in adult pancreas or during embryogenesis and that were poorly distributed in other compartments or developmental stages (data not shown). Among all 51 enriched genes in DPB, we selected 5 genes: Frem2 (Fras1-related extracellular matrix protein 2), which was not known to be expressed in the pancreas but it was annotated in the embryonic tissue [24]; Chst2 (carbohydrate sulfotransferase 2), whose expression was indicated both in the pancreas and in organogenesis [25]; Dsp (Desmoplakin), whose expression in the EST profile was not indicated either in the adult pancreas or in organogenesis, but it was required for the integrity of the mouse embryo [26,27]; Zim1 (Zinc finger, imprinted 1), an imprinted gene required during development [28]; and Wnk3 (WNK lysine-deficient protein kinase 3), whose EST profile indicated an expression in organogenesis and embryonic tissue but not in the adult pancreas.

**Figure 3:**
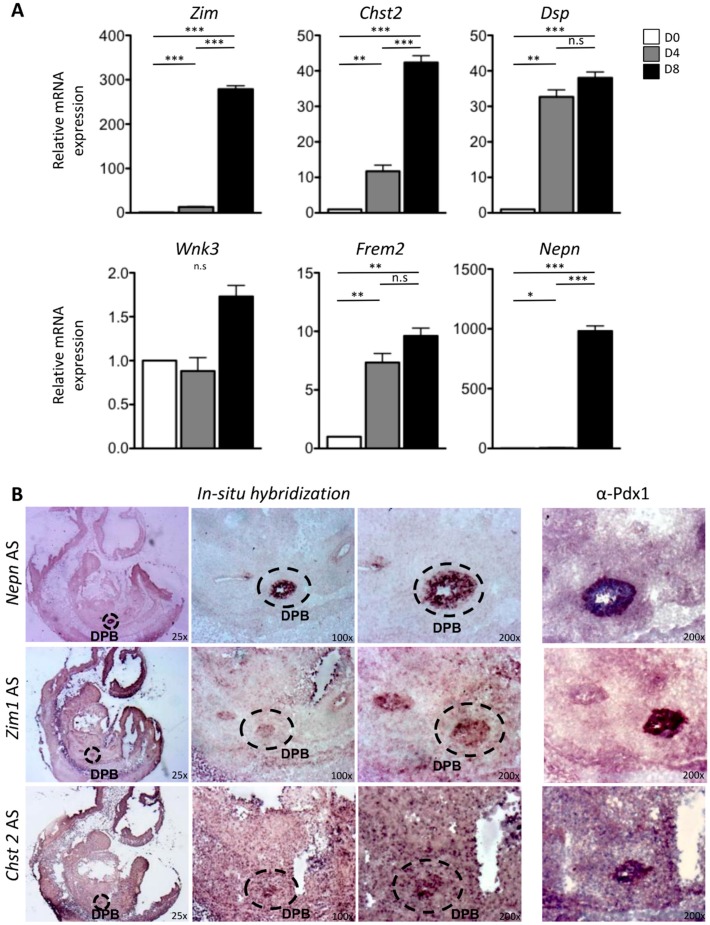
(**A**) qPCR of the selected enriched genes of DPB (Frem2, Chst2, Zim1, Dsp, and Wnk3) during mESCs differentiation. mESCs were plated at day 0 in a prodifferentiative medium supplemented with Matrigel and Activin A to induce the DE formation (D4). The cells were then treated with retinoic acid and FGF10 until the end of protocol to promote the Posterior Foregut Endoderm (PFE) formation. Total RNA was extracted at D0, D4, and D8 to perform qPCR. White, gray, and black histograms represent D0, D4, and D8, respectively. Nepn expression is used as bona fide marker for pancreatic differentiation. Gapdh expression level is used as reference gene. The data reported are normalized on Gapdh expression. Statistical analyses were performed using Student’s *t*-test, with *p* < 0.05 considered significant. (* *p* < 0.05, ** *p* < 0.01, *** *p* < 0.001, n.s *p* > 0.05). Values are shown as means of three independent RT-qPCR experiments ± SD in triplicates. (**B**) In situ hybridization of the selected DPB gene on mouse embryos at E10.5. In situ hybridization was performed on frozen sections with a probe recognizing Nepn (positive control), Zim1, and Chrst2 genes. Bud pancreas is positive for all genes tested. For each probe, three different magnifications are shown: 25×, 100×, and 200×. Right column shows IHC for Pdx1 on the same in situ slides. DPB: dorsal pancreatic bud (indicated by a circle). Data are representative of three independent experiments.

In order to validate their expression in pancreatic cells, we used ESCs to pancreatic differentiation as an in vitro model [3] (Figure S1). The expression levels of the selected five genes were analyzed by real-time PCR at day 0, day 4, and day 8 of the differentiation protocol. We used Nepn as a bona fide marker for pancreatic differentiation. Among the enriched genes of DPB, the expression of Chst2 and Zim1 had a strong enrichment at the posterior foregut endoderm (PFE) stage (D8) compared to D0 (mESCs) and D4 (definitive endoderm DE) (Figure 3A). More in detail, the expression of Zim1 was upregulated about 300 folds at D8 compared to D0 and the expression of Chst2 was upregulated approximately 40 folds at D8 compared to D0. Instead, the expression of Frem2, Wnk3, and Dsp was slightly upregulated at D8 compared to D4 and D0 (Figure 3A).

Therefore, to identify new putative markers of DPB, Zim1 and Chst2 that showed a significative upregulation at D8 were selected for the following analyses.

In order to evaluate whether the new enriched pancreatic candidates marked the bud at E10.5, we performed RNA in situ hybridization (ISH) on frozen E10.5 mouse embryo sections.

As shown in Figure 3B, the ISH experiment at E10.5 showed interesting results. Zim1 expression was detected in the epithelium of the DPB and in the posterior foregut cells, which give rise to the DPB showing an expression pattern very similar to Pdx1, comparing this result with immunohistochemistry data. On the other hand, Chst2 showed expression in several tissues including the pancreatic bud. In each experiment, we used Nepn antisense RNA as ISH-positive control. Thus, these data prove that Zim1 and Chst2 are novel embryonic pancreatic markers.

### 2.3. Validation of Enriched Intrinsic Factors and Characterization of Molecular Crosstalk between DPB and MeDPB at E10.5

To disclose the molecular crosstalk between the two districts during the primary transition phase of pancreatic organogenesis, the gene expression profiles of murine DPB and MeDPB at E10.5 were compared (Supplementary Table S1). Using a bioinformatics workflow, we analyzed the differential gene expression data to identify potential interactions across bud and mesenchyme molecules. We selected all secreted and cell-surface-bound products of analyzed transcriptome, based on Gene Ontology annotations, extracellular space (GO:0005615), and cell surface (GO:0009986) respectively. The interactions between secretory and receptor molecules were investigated querying STRING [29], one of the most accurate protein–protein associations database including both known and predicted interactions. We searched among secretory and receptor proteins significantly expressed in DPB or MeDPB for highest confidence score interactions (score ≥ 0.9). Each interaction was ranked on the basis of its potential involvement in bud and mesenchyme crosstalk. We assigned a priority score using three criteria: (i) mode of regulation–reverse regulation directions for two members of interaction (positive for member A and negative for member B or vice versa); (ii) size of regulation–fold change threshold significance (log2FC ≥ 1 or log2FC ≤ −1) for both members; (iii) cellular localization: cell surface for member A and extra-cellular space for member B, or vice versa. The highest score indicated the most interesting interactions occurring between a secreted molecule with a membrane receptor inversely expressed between two compartments Adopting these criteria, we obtained a list of 33 putative interacting molecules (16 DPB and 17 MeDPB) with the highest score characterized by a rank equal to 1 (Table 3).

**Figure 4:**
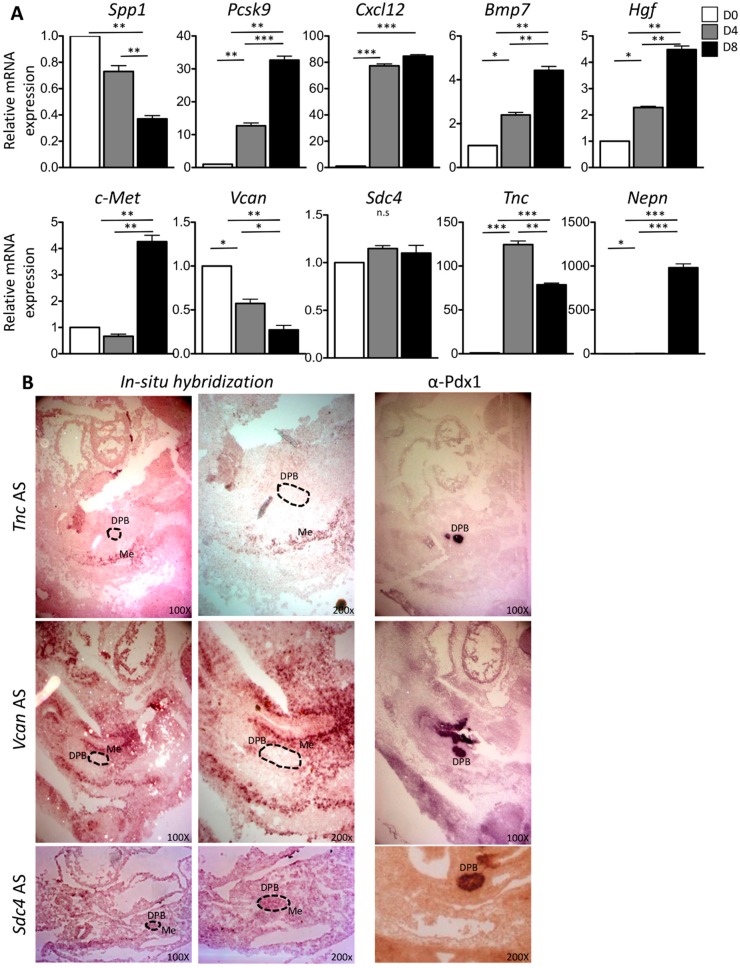
(**A**) RT qPCR of the selected novel candidate genes for DPB and MeDPB crosstalking (Spp1, Pcsk9, Cxcl12, Bmp7, Hgf, c-Met, Vcan, Sdc4, Tnc) during mESCs differentiations. Nepn expression is used as bona fide marker for pancreatic differentiation. Gapdh expression level is used as reference gene. The data reported are normalized on Gapdh expression. Statistical analyses were performed using Student’s *t*-test, with *p* < 0.05 considered significant. (* *p* < 0.05, ** *p* < 0.01, *** *p* < 0.001, n.s *p* > 0.05). Values are shown as means of three independent RT-qPCR experiments ± SD in triplicates. (**B**) In situ hybridization for candidate MeDPB genes on mouse embryos at E10.5. In situ hybridization was performed on frozen sections with a probe recognizing Vcan, Tnc, and Sdc4 genes. Vcan and Tnc are expressed in the mesenchyme surrounding pancreatic bud and *Sdc4* is expressed in the pancreatic bud. For each probe, two different magnifications are shown: 100× and 200×. Right column shows ISH for Pdx1 on the serial slides. DPB: dorsal pancreatic bud (indicated by dark circle), Me: mesenchyme. Data are representative of three independent experiments.

In order to evaluate whether newly selected DPB and MeDPB molecular crosstalk candidates could play a role during in vitro pancreatic differentiation, as previously, we used ESCs to pancreatic differentiation as an in vitro model [3]. The gene Spp1 showed a downregulation, suggesting that this gene was essentially expressed at the ESCs stage (D0). The Tnc was upregulated more than 100-fold at the DE stage (D4) and then slightly decreased at D8. The Pcsk9 was upregulated 34-fold at the PF stage (D8). The Cxcl12 showed an upregulation of more than 70-fold already at D4 of differentiation protocol. Bmp7, Hgf, and c-Met had a correlation with the in vitro pancreatic differentiation pattern but their expression levels were very low. The Sdc4 did not show any induction during the differentiation protocols (Figure 4A).

In order to validate our analysis, among the resulting interactions, we selected three genes: Sdc4 for DPB, and Vcan and Tnc for MeDPB.

As shown in Figure 4B, ISH experiments at E10.5 showed that Vcan and Tnc expressions were detected in the mesenchyme (Me) around the pancreatic bud area and Sdc4 at the pancreatic bud (DPB), comparing ISH results with Pdx1 immunohistochemistry data. These results confirmed that Vcan and Tnc were expressed in the mesenchyme surrounding the pancreatic bud. No ISH signal was detected for Vcan and Tnc in the DPB. The ISH result of Sdc4 displayed that although not exclusive, it is expressed in the pancreatic bud.

## 3. Discussion

Pancreatic organogenesis requires signals coming from the surrounding mesenchyme in order to define the proper morphogenesis of the epithelium. Despite several studies about the pathways governing this biological mechanism, the tight temporal signaling needs to be clarified. The lack of knowledge concerning the crucial role of the mesenchyme in the early pancreatic epithelium development has encouraged many research groups to investigate and describe the sequence of events essential to drive the pancreatic organogenesis from the early stages until the birth. In 2011, Landsman et al. performed the ablation of the pancreatic mesenchyme through diphtheria toxin employment in vivo, giving the first confirmation that the mesenchyme can sustain the pancreatic epithelium formation both at the early and late development stages [30]. Later, in 2013, Guo et al. demonstrated that the pancreatic mesenchyme, at E11.5, can activate signals to promote specific stem cells’ differentiation to pancreatic progenitors [31]. Furthermore, many studies have highlighted that the most important signaling pathways, contributing to the pancreas development, can be activated again in pathological conditions, such as during cellular damage in order to regenerate the tissue [32], in pancreatic cell necrosis with consequent fibrosis deposition [33], and in the pancreatic tumorigenesis process [34]. In addition, much evidence suggests the involvement of the same pathways in metastasis, by activating, in an aberrant manner in the adult tissue, the signaling required for the branching morphogenesis during pancreatic development [35]. All these considerations prompted us to dissect the mouse embryonic pancreas organogenesis, analyzing the contribution of novel genes in driving this process, by concert with signals from the mesenchyme.

By RNAseq analysis, we not only defined genes enriched in the DPB and MeDPB at E10.5, but using a bioinformatics workflow, the whole gene expression profiles of these two compartments were analyzed to disclose the molecular crosstalk between the two districts during the primary transition phase of pancreatic organogenesis. This approach allowed us to identify potential interactions across bud and mesenchyme molecules.

Among them, Spp1 has a different expression distribution during developmental stages and is also expressed in adult pancreas during regeneration processes [36]. Moreover, PCSK9 (proproteinconvertasesubtilisin/kexin type 9) is involved in pancreatic β-cell development and, in particular, in glucose metabolism by cleavage of pro-insulin to produce the insulin active form; the knockout mouse model for PCSK9 shows defects in pancreatic β-cell development. Furthermore, CXCL12 is a chemokine that binds the CXCR4 receptor activating a signaling pathway that plays a crucial role during embryogenesis and, in particular, in pancreatic organogenesis [37]; the axis CXCL12/CXCR4 is also involved in tissue repair [38]. Tenascin C (Tnc) is an extracellular matrix glycoprotein and, in the adult organism, is expressed during physiological and pathological conditions where cell migration and tissue remodeling are involved, like in the wound healing process [39]. Sdc4 is expressed in the murine foregut progenitors at E8.5 and E9.0, coordinating the activation of Wnt and BMP pathways during organogenesis [40], attesting to the involvement and the importance of this gene in foregut development. On the other hand, the pancreatic mesenchyme gene Vcan is an extracellular matrix protein and its expression is induced by TGF-β in cellular remodeling processes [41].

In our crosstalk analysis, the putative interaction between c-Met and HGF has been revealed, consistent with literature data [42]. It is very intriguing that c-MET/HGF signaling is a very well-studied pathway in tumorigenesis and metastasis, and could also play a crucial role during pancreas organogenesis.

Overall, we selected three genes as novel interesting candidates for DPB and MeDPB crosstalking: Vcan, Tnc and Sdc4. Vcan and Tnc encode ECM proteins that bind epithelial receptors (integrins) and other ECM molecules, such as fibronectin.

TNC is an ECM protein that, with its structure, is able to interact with a high number of highly diverse ligands. In the embryo, TNC is found in the outer mesenchyme and in several connective tissues, such as bone and cartilage [43]. TNC is known as an adhesion-modulating ECM protein that binds to fibronectin and it also binds cell surface receptors, like integrins [44], which interact with cytosolic components, such as actin-binding proteins [45], inducing cell adhesion and proliferation.

Vcan is expressed in the ECM of various tissues and organs, such as brain and breast. Vcan interacts with collagen and fibronectin of the ECM [46], promoting cell adhesion. Sdc4 has been shown to regulate adhesion assembly and cytoskeletal organization in response to fibronectin or other ECM proteins [47]. Modifications in Sdc4 sequence decrease RhoA activation and its related functions, such as adhesion and spreading, to promote cell migration [48]. In situ hybridization experiments showed that Tnc and Vcan are expressed in MeDPB.

In particular, the crosstalk between Sdc4 and Vcan is an intriguing interaction hypothesis between DPB and MeDPB because their contribution in cancer progression is known, playing a crucial role in mediating the EMT process [49,50].

Considering that those novel pancreas development biomarkers were also detected during pancreatic in vitro mESCs differentiation, our work opens future perspectives to further understand how to improve stem cell programming and reprogramming toward functional pancreatic cells. The improvement of differentiation protocol will lead to obtaining functional pancreatic cells, which could be used in cell replacement therapy after pancreatic injuries.

## 4. Materials and Methods

### 4.1. Ethics Statements

All animal experiments were performed in accordance with the regulations and guidelines of Italy and the European Union and were approved by the local ethical committee “Comitato Etico per la Sperimentazione Animale” (CESA) of IRSG. Biogem (384/2017-PR; 8 May 2017).

### 4.2. Mouse Strains and Husbandry

All mice were handled according to protocols approved by the Italian Ministry of Health. Mouse strain C57BL/6J (herein referred to as B6) was purchased from Charles River Laboratories. Animals were housed in an animal house under controlled conditions of temperature (22 ± 1 °C), humidity (55% ± 10%), and lighting on a 12 h light/12 h dark cycle, and were supplied with standard rodent food and water ad libitum.

Mice for testing were produced by crossing B6 females with B6 males. For tissue collections, all surgery was performed under anesthesia. All efforts were made to minimize suffering.

### 4.3. Embryo Dissection and Embedding for LCM

E10.5 embryos were obtained by crossing wildtype C57BL/6 mice. Embryonic age (E) was calculated by considering the morning when a vaginal plug was detected as E0.5. Embryos were dissected on ice under aseptic conditions in cold DEPC-treated phosphate-buffered saline (pH 7.2) (PBS-DEPC). After cryoprotection in 30% sucrose in PBSDEPC (overnight at 4 °C), embryos were embedded in OCT compound (Sakura, Zoeterwoude, The Netherlands) and stored at −80 °C.

### 4.4. Laser Capture Microdissection (LCM)

Tissue sections (8 μm) were cut with a cryostat (Leica Microm HM 500 M, Wetzlar, Germany) on polylysine slides (Menzel-Gläser, Braunschweig, Germany), stored on dry ice (1 h), and stained with eosin (70% ethanol 30 s, dH2O DEPC 20 s, 70% ethanol 20 s, 95% ethanol 20 s, eosinY 2 s, 95% ethanol 10 s, 95% ethanol 10 s, 100% ethanol 30 s, 100% ethanol 60 s, xylene 5 min, drying 5 min). LCM was performed immediately thereafter using the Palm Zaiss system (Palm Zaiss, Jena, Germany) under a 20× objective. Pancreas bud and surrounding mesenchyme were captured on plastic 0.5 mL tube. Pancreas bud was identified using immunohistochemistry for Pdx1 on serial section of ones used for dissecting while the mesenchyme dissection was done using an algorithm to cut at a regular distance between DPB and surrounding tissue.

Six B6 embryos at E10.5 were used to dissect DPB and MeDPB. Three dissected DPB and three dissected MeDPB were pooled together to generate the two biological replicates used for RNAseq.

### 4.5. RNA Isolation and RNAseq

Total RNA from cells obtained by LCM was isolated using the Pico-Pure RNA isolation kit (Arcturus, San Diego, CA, USA). RNA quality and integrity were determined using Agilent Bioanalyzer (Agilent Technologies, Santa Clara, CA, USA) on RNA 6000 Pico Lab-Chip Kit. Two biological replicates with RNA integrity numbers (RIN) higher than 8.5 for DPB and MeDPB were considered to prepare libraries with SMART-Seq Ultra Low Input RNA Kit. All biological replicates were normalized to the same input amount of total RNA. All libraries were multiplexed and sequenced with Illumina HiSeq2500 as 100-nucleotide paired-end reads.

### 4.6. RT-qPCR Analysis

cDNA was generated with the SuperScript^®^ III First-Strand Synthesis System for RT-PCR (Invitrogen 18080051), according to the manufacturer’s specifications. A total of 1 μg of total RNA was used for each cDNA synthesis. Primer 3 software (http://primer3.ut.ee/) was used to design the oligo primers setting the annealing temperature to 59–61 °C for all primer pairs. Oligo sequences are reported in Supplementary Table S4.

For gene expression analyses, the same amount of cDNA was used for each PCR reaction with each primer pair (forward/reverse primers mix: 0.2 μM, in a final volume of 25 μL). Real-time PCR analysis was performed using the iTaq™ Universal SYBR^®^ Green Supermix (BIORAD, Hercules, CA, USA) in a 7500 Real-Time PCR System (Applied Biosystems, Foster City, CA, USA) under the following conditions: 2 min at 50 °C, 10 min at 95 °C, followed by 40 cycles of 15 s at 95 °C and 1 min at 60 °C. The Gapdh mRNA expression was used as a control to normalize the data. The gene expression experiments were performed in triplicate in three independent experiments and a melting analysis was performed at the end of the PCR run to confirm gene-specific amplification. To calculate the relative expression levels, we used the 2^−ΔΔ*C*t^ method [51,52]. Comparison of data sets was performed by Student’s *t*-test and a value of *p* < 0.05 was considered significant [53].

PCR amplification efficiency was calculated for Zim1, Chst2, Vcan, Sdc4, Tnc, and Gapdh, using serial dilutions of cDNA (1:1; 1:10; 1:100, and 1:1000). Each point was analyzed in duplicate two times on two different 96-well plates (Plate 1 and Plate 2). For the analysis, only *C*t data present at least two times were considered. *C*t values were plotted on the y-axis along with corresponding logarithmic dilutions (x-axis). A linear regression curve and slope were determined for each gene using Microsoft Office software. Amplification efficiency was calculated using the equation: E = −1 + 10^(−1/slope)^ (Supplementary Figure S2 and Table S5).

### 4.7. ESCs Culture and Differentiation

ESCs were incubated at 37 °C in 5% CO_2_; medium was changed daily and cells were split every 2–3 days routinely. For the endoderm differentiation, 5 × 10^4^/plate cells were seeded in 35 mm dishes, as a feeder-free monolayer. The differentiation medium consisted of DMEM Low Glucose (Lonza, Valais, Switzerland), supplemented with 5% FBS (HyClone, Logan, UT, USA), 2 mM l-Glutamine (Gibco, Dublin, Ireland), 1 mM nonessential amino acids (Gibco), 100 U-μg/mL Penicillin/Streptomycin (Gibco), 0.1 mM β-Mercaptoethanol (Sigma, St. Louis, MO, USA), depleted of LIF, with 200 μg/mL Matrigel (BD Biosciences, Qume Drive, San Jose, CA, USA) and treated sequentially with several molecules at different time points for eight days. These factors included activin A (20 ng/mL, R&D Systems, Minneapolis, MN, USA), all trans retinoid acid (RA, 5 μM, Sigma), fibroblast growth factor 10 (Fgf10, 10 ng/mL, R&D Systems), cyclopamine (CYC, 10 μM, Sigma), *N*-*N*-(3,5-difluorophenacetyl)-Lalanylsphenylglycinet- butylesterm (DAPT, 5 μM, Sigma). Medium was changed every two days [3].

### 4.8. In Situ Hybridization

E10.5 embryos from matings of B6 mice were fixed in 4% paraformaldehyde (overnight, 4 °C), cryoprotected in 30% sucrose in PBS (overnight, 4 °C), embedded in OCT (Sakura), quick-frozen over dry ice/ethanol slurry, and stored at −80 °C. Frozen sections (10 μm) were obtained as described above on Superfrost slides (Mentzel-Gläser, Saarbrückener, Germany). Templates for riboprobes were generated by PCR from commercially available plasmids (Open Biosystems, Huntsville, AL, USA or Gene Service, Cambridge, UK) or from cDNA obtained by reverse transcription of mRNA from E10.5 mouse embryos using forward and reverse primers extended at their 5′ ends with either T7 or SP6 promoter sequences (T7. GGATTTAATACGACTCACTATAGGGAGA; Sp6. CGATTTAGGTGACACTATAGA), following a protocol from the GenePaint consortium (http://www.genepaint.org) (7 min initial template denaturation at 95 °C, 35 cycles with 1 min denaturation (95 °C), 1 min primer annealing (52–68 °C), 1 min elongation (72 °C), final elongation for 7 min at 72 °C). The resulting PCR products were resolved on agarose gels and the right bands excised and purified using the QIAGEN gel extraction kit (QIAGEN, Hilden, Germany). After PCR reamplification, products were purified using QIAquick PCR purification kit (QIAGEN), analyzed on an agarose gel, and verified by sequencing. Nepn antisense riboprobe was a 743 bp used as a positive control. Digoxygenin-labeled riboprobes (sense and antisense) were obtained using a DIG-labeling RNA kit (Roche Diagnostics Basel, Switzerland) following the manufacturer’s instructions. Mouse embryos at E10.5 were cut into 7 μm frozen sections that were collected on Thermo Scientific™ SuperFrost Plus™ adhesion slides. In situ hybridization was performed as described in Fagman et al. (2011) [54,55]. Images were processed using the Axion Vision software and Image J software. Oligos used to amplify the in situ probe are illustrated in Supplementary Table S5. All in situ probes were about 750 bp. Oligo sequence used to amplify gene specific probes are described in Supplementary Table S4.

### 4.9. Immunohistochemistry

Paraffin sections, 10 μm thick, were collected on polylysine glass slides [56].

The incubation with the primary antibodies for Pdx1 (ab47267 rabbit) was performed overnight at 4 °C in histoblock solution (3% BSA, 5% NGS, 20 mM, MgCl_2_, 0.3% Tween 20 in PBS, pH 7.2); staining procedures and chromogenic reactions were carried out according to the protocols of the Vectastain ABC kit and “DAB substrate kit for peroxidase” (Vector Laboratories, Burlingame, CA, USA). Images were obtained using an Axioplan2 microscope equipped with an Axiocam digital camera (Zeiss, Oberkochen, Germany) and processed using the Axion Vision software. The digital images were assembled into composite pictures using Adobe Photoshop (Adobe Systems, San Jose, CA, USA).

### 4.10. Statistics in Biological Experiments

Where data were averaged, the samples stemmed from independent experiments with independent preparations; that is, they represent biological replicates [57]. Significance of differences has been evaluated through Student’s *t*-test. Differences are only mentioned and interpreted as such if *p* < 0.005.

### 4.11. RNAseq Data Analysis

Sequencing quality was assessed through error rate and base quality distributions of reads for each sample. We filtered the raw data removing reads containing adaptors, reads containing more than 10% of bases that could not be determined, reads including over 50% bases with a Phred quality score ≤ 5. The reads were aligned to human reference genome (GRCh37/hg19) using TopHat [58] and the expression was quantified at gene level using featureCounts [59]. Downstream analysis of gene expression was performed in the R statistical environment by fitting a negative binomial model and estimating dispersion using edgeR [60]. Differentially expressed genes between DPB and MeDPB were identified using the exact test based on the qCML methods. Exact *p*-value was computed by summing overall sums of counts that had a probability less than the probability under the null hypothesis of the observed sum of counts. Differentially expressed genes were selected by absolute log2FoldChange ≥ 1 and False Discovery Rate (FDR) adjusted *p*-value ≤ 0.05. Gene Ontology (GO) enrichment was computed using over-representation test and FDR correction (FDR-adjusted *p*-value ≤ 0.05).

Data availability statement: Authors declare to make materials. Data and associated protocols promptly available to readers without undue qualifications in material transfer agreements.

## Figures and Tables

**Table 1:** List of the enriched DPB genes in mouse embryo at E10.5.

Gene Name	CPM DPB Mean	Gene Name	CPM DPB Mean
*Peg3*	3769.3	*Fryl*	187.7
*Rian*	728.7	*Zim1*	167.5
*Nepn*	686.2	*Rap1gap2*	167.0
*Ptprf*	638.4	*Etl4*	161.1
*Gcg*	571.5	*Itga6*	159.2
*Meg3*	567.0	*Lama5*	157.4
*Myh9*	376.5	*Epcam*	155.1
*Frem2*	369.1	*Nav2*	154.0
*Fras1*	356.4	*Appl2*	153.5
*Ccnd1*	322.5	*Nr5a2*	152.6
*Prox1*	308.0	*Svil*	143.9
*Spon1*	303.3	*Parm1*	135.8
*Cdh1*	291.1	*Itpr3*	135.7
*Ptpn13*	281.9	*Abcc8*	135.5
*Slc38a5*	275.5	*Plk2*	129.4
*Dlk1*	266.3	*Fbxo21*	128.0
*Chst2*	243.9	*Wnk3*	127.6
*Adamts1*	230.2	*Lrba*	120.0
*Dlg5*	229.1	*Arg1*	119.5
*Dsp*	225.8	*Wnk2*	118.5
*Notch1*	214.6	*Jmy*	114.6
*Sox9*	210.0	*Cep170b*	113.2
*Ankrd50*	189.5	*Rnf213*	111.9
*Pam*	189.4	*Fndc3b*	110.4
*Aes*	188.9	*Gatsl2*	109.5

**Table 2:** List of the enriched MeDPB genes in mouse embryo at E10.5.

Gene Name	CPM MeDPB Mean	Gene Name	CPM MeDPB Mean
*Mest*	589.9	*Akap12*	85.67
*Fbn2*	401.2	*Efnb2*	83.16
*Zfp462*	352.5	*Oxct1*	80.50
*Zfhx4*	217.0	*Epha4*	78.03
*Sulf2*	164.1	*Zfp423*	77.27
*Cdh11*	157.5	*Fbn1*	75.36
*Dpysl3*	157.0	*Sacs*	75.36
*Nefm*	144.0	*Arhgef40*	71.37
*Fndc3c1*	140.0	*Aff3*	70.69
*Slit2*	131.7	*Runx1t1*	67.79
*Basp1*	122.1	*Lin28b*	67.29
*Gli3*	117.3	*Dennd5b*	66.06
*Nid1*	116.7	*Cachd1*	64.35
*Cald1*	111.8	*Kdr*	64.18
*Amot*	108.9	*Phf6*	64.12
*Msn*	105.8	*Nefl*	62.98
*Zeb2*	101.9	*Foxp2*	62.41
*2810417H13Rik*	95.3	*Map1a*	60.62
*Hdgfrp3*	92.8	*Ets1*	60.32
*Nhsl2*	90.4	*Far1*	59.52
*Flnc*	88.6	*Col11a1*	55.89
*Col5a1*	88.5	*Mmp2*	55.79
*Phactr2*	87.8	*Cdh5*	55.58
*Hmcn1*	86.9	*Atp11c*	55.41
*Pcdh18*	86.7	*Adam19*	55.25

**Table 3:** List of the crosstalk between pancreatic bud and mesenchyme. According to their scores (rank = 1), we obtained putative crosstalking interactions (16 bud and 17 mesenchymal proteins).

Genes Expressed in Pancreatic Bud	
**Gene Name**	**Description**
*Gcg*	glucagon [Source:MGI Symbol;Acc:MGI:95674]
*Bmp7*	bone morphogenetic protein 7 [Source:MGI Symbol;Acc:MGI:103302]
*Met*	met proto-oncogene [Source:MGI Symbol;Acc:MGI:96969]
*Spp1*	secreted phosphoprotein 1 [Source:MGI Symbol;Acc:MGI:98389]
*Pcsk9*	proproteinconvertasesubtilisin/kexin type 9 [Source:MGI Symbol;Acc:MGI:2140260]
*Clu*	clusterin [Source:MGI Symbol;Acc:MGI:88423]
*Pyy*	peptide YY [Source:MGI Symbol;Acc:MGI:99924]
*Edn3*	endothelin 3 [Source:MGI Symbol;Acc:MGI:95285]
*Sdc4*	syndecan 4 [Source:MGI Symbol;Acc:MGI:1349164]
*Serpinf2*	serine (or cysteine) peptidase inhibitor, clade F, member 2 [Source:MGI Symbol;Acc:MGI:107173]
*Pcsk6*	proproteinconvertasesubtilisin/kexin type 6 [Source:MGI Symbol;Acc:MGI:102897]
*F5*	coagulation factor V [Source:MGI Symbol;Acc:MGI:88382]
*Iapp*	islet amyloid polypeptide [Source:MGI Symbol;Acc:MGI:96382]
*Casr*	calcium-sensing receptor [Source:MGI Symbol;Acc:MGI:1351351]
*Cck*	cholecystokinin [Source:MGI Symbol;Acc:MGI:88297]
*Sct*	secretin [Source:MGI Symbol;Acc:MGI:99466]
**Gene Name**	**Description**
*Vcan*	versican [Source:MGI Symbol;Acc:MGI:102889]
*Tnc*	tenascin C [Source:MGI Symbol;Acc:MGI:101922]
*Igf1*	insulin-like growth factor 1 [Source:MGI Symbol;Acc:MGI:96432]
*Cxcl12*	chemokine (C-X-C motif) ligand 12 [Source:MGI Symbol;Acc:MGI:103556]
*Tgfb2*	transforming growth factor, beta 2 [Source:MGI Symbol;Acc:MGI:98726]
*Thbs1*	thrombospondin 1 [Source:MGI Symbol;Acc:MGI:98737]
*Itga8*	integrin alpha 8 [Source:MGI Symbol;Acc:MGI:109442]
*Lpar1*	lysophosphatidic acid receptor 1 [Source:MGI Symbol;Acc:MGI:108429]
*Pcsk5*	proproteinconvertasesubtilisin/kexin type 5 [Source:MGI Symbol;Acc:MGI:97515]
*Apob*	apolipoprotein B [Source:MGI Symbol;Acc:MGI:88052]
*Adcyap1r1*	adenylate cyclase activating polypeptide 1 receptor 1 [Source:MGI Symbol;Acc:MGI:108449]
*Cxcr4*	chemokine (C-X-C motif) receptor 4 [Source:MGI Symbol;Acc:MGI:109563]
*Dcn*	decorin [Source:MGI Symbol;Acc:MGI:94872]
*Thbd*	thrombomodulin [Source:MGI Symbol;Acc:MGI:98736]
*Hgf*	hepatocyte growth factor [Source:MGI Symbol;Acc:MGI:96079]
*Ntsr1*	neurotensin receptor 1 [Source:MGI Symbol;Acc:MGI:97386]
*Bmp2*	bonemorphogeneticprotein2 [Source:MGI Symbol;Acc:MGI:88177]

## Supplementary Materials

Supplementary materials can be found at https://www.mdpi.com/1422-0067/20/19/4900/s1. Supplementary Table S1. List of 1744 differentially expressed genes. Among the significative differently expressed genes in our RNAseq analysis (log2FC ≥ 4 and log2FC ≤ −4), we selected genes that were reported in literature as expressed in pancreatic bud and mesenchyme, respectively. In red are reported 931 genes specifically enriched in DPB and in green 813 genes specifically enriched in MeDPB; Supplementary Table S2. Functional Enrichment Analysis on DPB enriched genes; Supplementary Table S3. Functional Enrichment Analysis on MeDPB enriched genes; Supplementary Table S4. Oligo’s list used for RT-qPCR and in-situ hybridization probes; Supplementary Table S5. Amplification Efficiency data; Supplementary Figure S1. Schematic protocol of directed differentiation from mESCs into PFE. ESCs were plated at 50.000 cells/cm2 at day 0 (M0) in a prodifferentiative medium supplemented with Matrigel (200 μg/mL) and Activin A (20 ng/mL) to induce the DE formation (M1). The cells were then treated with retinoic acid (5 μM) and FGF10 (10 ng/mL) until the end of protocol to promote the Posterior Foregut Endoderm (PFE) formation (M2).

## Abbreviations

B6	C57BL/6J
BP	Biological Processes
CC	Cellular components
CESA	Comitato Etico per la Sperimentazione Animale
Chst2	Carbohydrate sulfotransferase 2
CPM	Counts per millions
DE	Definitive endoderm
DPB	Dorsal pancreatic bud
Dsp	Desmoplakin
E10.5	Embryonic day 10.5
ESCs	Embryonic stem cells
EST	Expressed Sequence Tag
FDR	False Discovery Rate
Frem2	Fras1-related extracellular matrix protein 2
GO	Gene ontology
IHC	Immunohistochemistry
ISH	In situ hybridization
iPSCs	Induced pluripotent stem cells
LCM	Laser capture microdissection
MeDBP	DPB mesenchyme
mESCs	Mouse embryonic stem cells
MO	Molecular functions
PBS-DEPC	DEPC-treated phosphate-buffered saline
PCA	Principal component analysis
PCSK9	Proproteinconvertasesubtilisin/kexin type 9
PFE	Posterior foregut endoderm
PPCs	Pancreatic progenitor cells
RA	Retinoic acid
RIN	RNA integrity numbers
RNAseq	RNA high-throughput sequencing
Sdc4	Syndecan 4
Tnc	Tenascin C
Vcan	Versican
Wnk3	WNK lysine-deficient protein kinase 3
Zim1	Zinc finger, imprinted 1
