# Supplementary material for: Exploring the Molecular Crosstalk between Pancreatic Bud and Mesenchyme in Embryogenesis: Novel Signals Involved
Source: Int J Mol Sci. 2019 Oct 3;20(19):4900. doi: 10.3390/ijms20194900 (PMC6811752; doi:10.3390/ijms20194900)
Supplement: Supplementary file 1 [file ijms-20-04900-s001.zip › ijms-543005-supplementary/Supplementary Figure S1.pdf]

Supplementary Figure S1

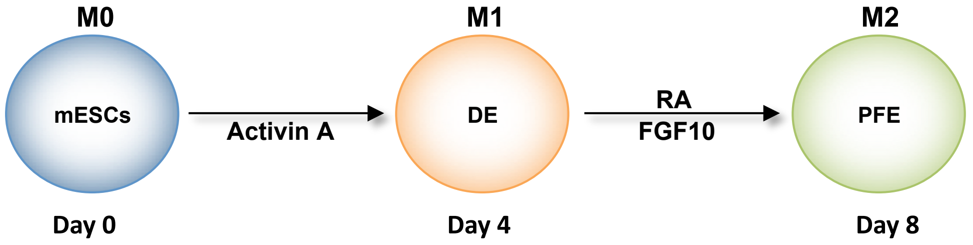

Supplementary Figure S1: Schematic protocol of directed differentiation from mESCs into PFE. ESCs were plated at 50.000 cells/cm2 at day0 (M0) in a pro differentiative medium supplemented with Matrigel (200µg/ml) and Activin A (20ng/ml) to induce the DE formation (M1). The cells were then treated with Retinoic Acid (5µM) and FGF10 (10ng/ml) until the end of protocol to promote the Posterior Foregut Endoderm (PFE) formation (M2).
