# Supplementary material for: Exploring the Molecular Crosstalk between Pancreatic Bud and Mesenchyme in Embryogenesis: Novel Signals Involved
Source: Int J Mol Sci. 2019 Oct 3;20(19):4900. doi: 10.3390/ijms20194900 (PMC6811752; doi:10.3390/ijms20194900)
Supplement: Supplementary file 1 [file ijms-20-04900-s001.zip › ijms-543005-supplementary/Supplementary Figure S2.pdf]

Supplementary Figure S2: Amplification Efficiency

Gene of interest: *Zim1*

GOI: Zim1	Replicates (Ct)				Ct Average	sd	Dilution	Log Dilution	Ct Average
Zim1 1:1	28,07	27,86	27,27	27,77	27,74	0,34	1,00	0,00	27,74
Zim1 1:10	31,17	31,55	31,15	31,87	31,44	0,34	0,10	-1,00	31,44
Zim1 1:100	33,79	34,93	33,74	33,71	34,04	0,59	0,01	-2,00	34,04
Zim1 1:1000	38,42	38,16	38,57	nd	38,38	0,21	0,00	-3,00	38,38

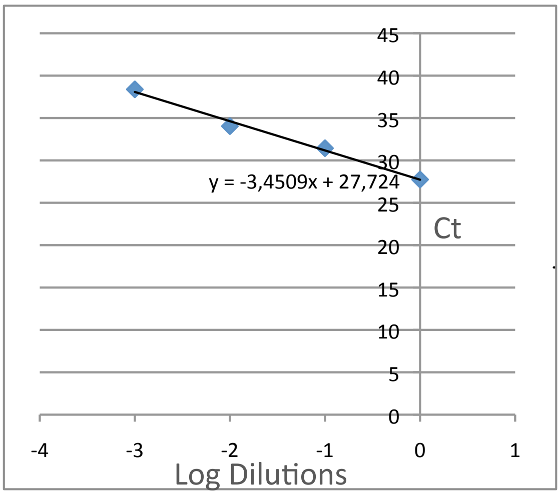

**Slope**		**-3.45**
**Amplification factor**		**1.95**
**Efficiency**		**95%**

Gene of interest: *Chst2*

GOI: Chst2	Replicates (Ct)				Ct Average	sd	Dilution	Log Dilution	Ct Average
Chst2 1:1	29,55	29,00	29,48	29,31	29,34	0,25	1,00	0,00	29,34
Chst2 1:10	32,09	32,22	32,15	33,92	32,60	0,89	0,10	-1,00	32,60
Chst2 1:100	nd	36,64	35,73	36,43	36,27	0,47	0,01	-2,00	36,27
Chst2 1:1000	nd	35,83							

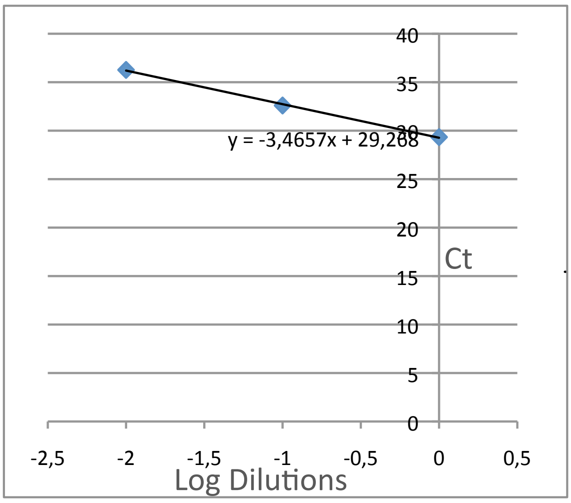

**Slope**		**-3.46**
**Amplification factor**		**1,95**
**Efficiency**		**95%**

Amplification Efficiency

Gene of interest: *Vcan*

GOI:Vcan	Replicates (Ct)				Ct Average	sd	Dilution	Log Dilution	Ct Average
Vcan 1:1	24,08	23,50	24,14	23,91	23,91	0,29	1,00	0,00	23,91
Vcan 1:10	27,26	27,16	27,07	27,68	27,29	0,27	0,10	-1,00	27,29
Vcan 1:100	30,60	30,84	31,46	30,88	30,94	0,36	0,01	-2,00	30,94
Vcan 1:1000	34,34	33,79	33,92	33,57	33,90	0,32	0,00	-3,00	33,90

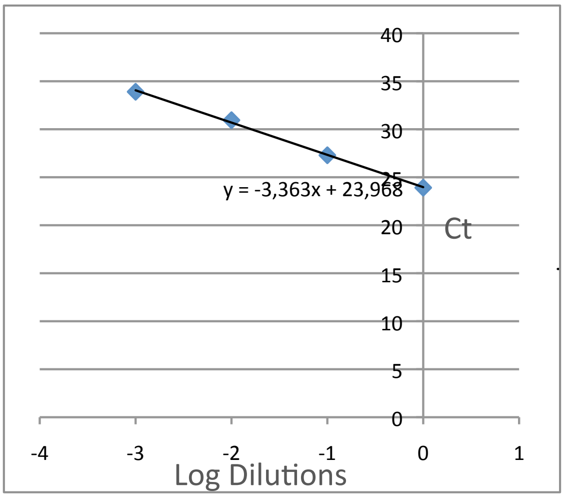

Slope		-3.36
Amplification factor		1.98
Efficiency		98%

Gene of interest: *Sdc4*

GOI:Sdc4	Replicates (Ct)				Ct Average	sd	Dilution	Log Dilution	Ct Average
Sdc4 1:1	26,56	25,95	26,48	25,98	26,24	0,32	1,00	0,00	26,24
Sdc4 1:10	30,83	28,95	30,23	29,62	29,91	0,81	0,10	-1,00	29,91
Sdc4 1:100	32,72	32,28	33,22	33,55	32,94	0,56	0,01	-2,00	32,94
Sdc4 1:1000	35,70	36,29	nd	36,94	36,31	0,62	0,00	-3,00	36,31

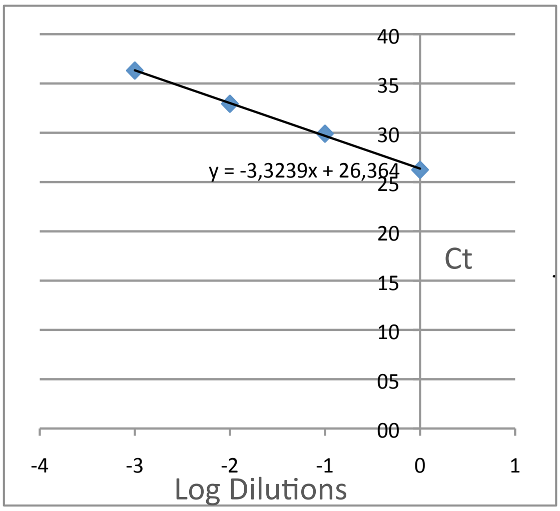

Slope		-3.32
Amplification factor		2
Efficiency		100%

Amplification Efficiency

Gene of interest: *Tnc*

GOI: Tnc	Replicates (Ct)				Ct Average	sd	Dilution	Log Dilution	Ct Average
Tnc 1:1	28,37	27,80	27,84	27,72	27,93	0,30	1,00	0,00	27,93
Tnc 1:10	32,34	31,36	31,25	32,06	31,75	0,53	0,10	-1,00	31,75
Tnc 1:100	35,68	34,68	35,10	34,17	34,91	0,64	0,01	-2,00	34,91
Tnc 1:1000									

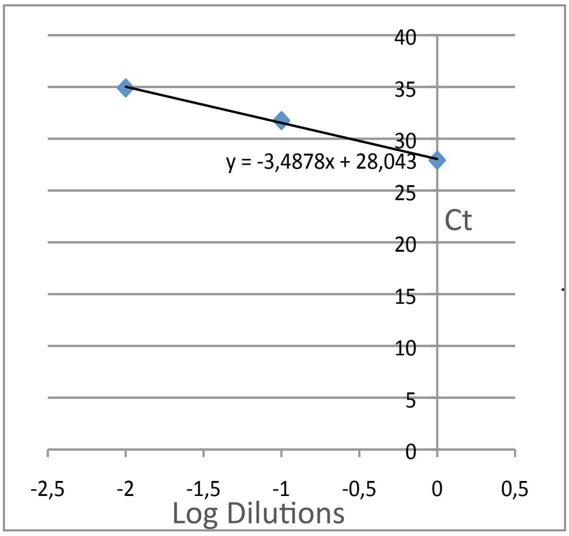

**Slope**		**-3.48**
**Amplification factor**		**1.94**
**Efficiency**		**94%**

Reference Gene: *Gapdh*

RG: Gapdh	Replicates (Ct)				Ct Average	sd	Dilution	Log Dilution	Ct Average
Gapdh 1:1	20,75	20,55	21,47	20,42	20,80	0,47	1,00	0,00	20,80
Gapdh 1:10	23,75	23,95	24,16	24,22	24,02	0,22	0,10	-1,00	24,02
Gapdh 1:100	27,45	27,09	27,31	27,51	27,34	0,19	0,01	-2,00	27,34
Gapdh:1000	30,92	30,09	31,54	30,53	30,77	0,62	0,00	-3,00	30,77

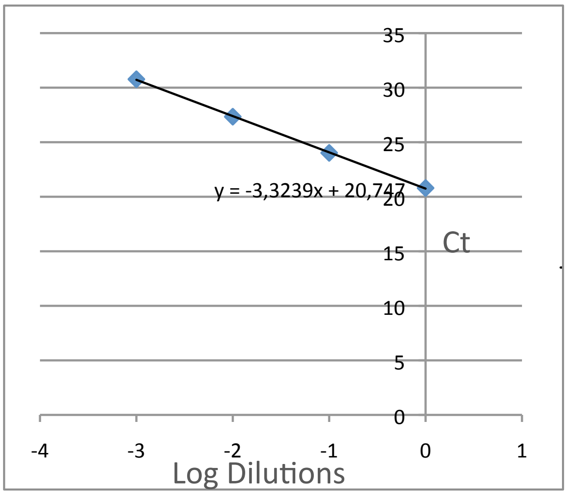

**Slope**		**-3.32**
**Amplification factor**		**2**
**Efficiency**		**100%**

Supplementary Figure S2: PCR Amplification Efficiency.

Serial diluitions of cDNA (1:1; 1:10; 1:100 and 1:1000) were used to performed amplification efficiency for *Zim1, Chst2, Vcan, Sdc4, Tnc* genes and for the reference gene *Gapdh.* Ct values were plotted on the y-axis along with corresponding logarithmic dilutions (x-axis). A linear regression curve and slope was determined for each genes. Amplification efficiency was calculated using the equation: $E = -1+10^{(-1/slope)}$. Each point was analyzed in duplicate two times on two different 96-well plates.
