# Supplementary material for: Exploring the Molecular Crosstalk between Pancreatic Bud and Mesenchyme in Embryogenesis: Novel Signals Involved
Source: Int J Mol Sci. 2019 Oct 3;20(19):4900. doi: 10.3390/ijms20194900 (PMC6811752; doi:10.3390/ijms20194900)
Supplement: Supplementary file 1 [file ijms-20-04900-s001.zip › ijms-543005-supplementary/Supplementary table S4.docx]

**Supplementary Table** S**4**: Oligo’s list used for RT-qPCR and in-situ hybridization probes

| **Oligo name** | **Sequence 5’ to 3’** |
| --- | --- |
| Spp1 Fw RT | TGCACCCAGATCCTATAGCC |
| Spp1 Rev RT | CTCCATCGTCATCATCATCG |
| Pcsk9 Fw RT | CCTTGGAGGTTCAAAGACCA |
| Pcsk9 Rev RT | CTGGAGGTGTGCAGAACTGA |
| Hgf Fw RT | AGGAACAGGGGCTTTACGTT |
| Hgf Rev RT | GTCAAATTCATGGCCAAACC |
| Met Fw RT | TCCTGCACTGTGAGCATTTC |
| Met Rev RT | ACGTAATGAGGGTGGAGTGC |
| Vcan Fw RT | ACCAAGGAGAAGTTCGAGCA |
| Vcan Rev RT | CTTCCCAGGTAGCCAAATCA |
| BMP7 FW RT | CGAGACCTTCCAGATCACAGT |
| BMP7 REV RT | CAGCAAGAAGAGGTCCGACT |
| Cxcl12 Fw RT | TAAAGCCCTTCATGGTCCTG |
| Cxcl12 Rv RT | AGTCCAGCCTGCTATCCTCA |
| Tnc Fw RT | TGGAGTACGAGCTGCATGAC |
| Tnc Rv RT | AAACTTGGTGGCGATGGTAG |
| Frem2 Fw RT | CAGGATGCTTAGAGCCTTGG |
| Frem 2 Rv RT | GTCCTTGAGCACCTTTGAGC |
| Chst2 Fw RT | CGTGACATTTAGCTGCCAGA |
| Chst2 Rv RT | CATACACGCTGCCTCAGAAA |
| Dsp Fw RT | CTGGGTACCTGCCAAGATGT |
| Dsp Rv RT | CGTTGATTTTCACGGTGATG |
| Wnk3 Fw RT | AACGCCTTCGAGCAACTAAA |
| Wnk3 Rv RT | GGCTTCGAAGTTTGCTTTTG |
| Zim1 Fw RT | CGGGCAATAAGGAGAATGAA |
| Zim Rv RT | GGTCTGTGGTTTTCCCTCAA |
| Fndc3c1 Fw RT | GCCGGATCTCTTGCTATCTG |
| Fndc3c1 Rv RT | ATGGGCTCTCCATTGATGAC |
| Zfhx4 Fw RT | TTTTGCAAGAAGCCTCCAGT |
| Zfhx4 Rv RT | AAGGTCGAGCTTTGGCTGTA |
| Sdc4 Fw RT | CTGATCCTGCTGCTGGTGTA |
| Sdc4 Rv RT | GGAGGAAGCTTCATGCGTAG |
| Zim1 SP6 | TCAAGCCAAAGTTGATCACCAGGTTG |
| Zim1 T7 | AGAGTGGAGCTGCTGTTGAAGG |
| Chst2 SP6 | TGCCCTGAACATGACCAGTGG |
| Chst2 T7 | AGAGTGGAGCTGCTGTTGAAGG |
| Nepn Sp6 | TACATGAGCATGGAGAACAACC |
| Nepn T7 | CTGGCTCGTAGGAGACTGCAA |
| Vcan Sp6 | AACCACAAACTCCAGGGAGCCC |
| Vcan T7 | GGTCCCTGCTGCAGGTGTTGAA |
| Tnc Sp6 | TTGAGCCCAGGCAGGTCCACT |
| Tnc T7 | TGATCCAGCAACCATCAATGCG |
| Sdn4 Sp6 | ACAGCCACACGAAAACTCACGC |
| Sdn4 T7 | TACTGTGTGAATCCCGGCTCC |

Note:

For in-situ hybridization oligos, Sp6 and T7 sequence are added to the oligo at 5’ position

Sp6 sequence: CGATTTAGGTGACACTATAGA

T7 sequence: GATTTAATACGACTCACTATAGGGAGA
